# The Principles, Mechanisms, and Benefits of Unconventional Agents in the Treatment of Biofilm Infection

**DOI:** 10.3390/ph13100299

**Published:** 2020-10-10

**Authors:** Jasminka Talapko, Ivana Škrlec

**Affiliations:** Faculty of Dental Medicine and Health, Josip Juraj Strossmayer University of Osijek, HR-31000 Osijek, Croatia; jtalapko@fdmz.hr

**Keywords:** antimicrobial peptides, bacteriophage, biofilm, immunomodulatory action, resistance

## Abstract

Today, researchers are looking at new ways to treat severe infections caused by resistance to standard antibiotic therapy. This is quite challenging due to the complex and interdependent relationships involved: the cause of infection–the patient–antimicrobial agents. The sessile biofilm form is essential in research to reduce resistance to very severe infections (such as ESKAPE pathogens: *Enterococcus faecium, Staphylococcus aureus, Klebsiella pneumoniae, Acinetobacter baumanni, Pseudomonas aeruginosa,* and *Enterobacter spp*). The purpose of this study is to elucidate the mechanisms of the occurrence, maintenance, and suppression of biofilm infections. One form of biofilm suppression is the efficient action of natural antagonists of bacteria—bacteriophages. Bacteriophages effectively penetrate the biofilm’s causative cells. They infect those bacterial cells and either destroy them or prevent the infection spreading. In this process, bacteriophages are specific, relatively easy to apply, and harmless to the patient. Antimicrobial peptides (AMPs) support the mechanisms of bacteriophages’ action. AMPs could also attack and destroy infectious agents on their own (even on biofilm). AMPs are simple, universal peptide molecules, mainly cationic peptides. Additional AMP research could help develop even more effective treatments of biofilm (bacteriophages, antibiotics, AMPs, nanoparticles). Here, we review recent unconventional agents, such as bacteriophages and AMPs, used for eradication of biofilm, providing an overview of potentially new biofilm treatment strategies.

## 1. Introduction

The natural course of human life, from its beginning until the present day, has been marked by transition, and it is still passing through often unknown processes of adaptation and evolution [1]. Destroying life, and even destroying the smallest carriers—microorganisms, is not easy, and very often impossible [2]. Is it possible at all, and for how long, to delay the unfavorable and unwanted course of an event? The ineffectiveness of antibacterial drugs is not an isolated phenomenon, but an increasingly common occurrence [3,4,5]. Increasing bacterial resistance is connected to patient and clinicians’ malpractice in prescribing and using antibiotics [6].

When science created the first antibiotics preparations, to facilitate and raise the quality of human life, humanity was relieved [7]. Deadly diseases became transient conditions, and a growing selection of effective drugs guaranteed an optimistic future and extended life expectancy [8]. Simultaneously, the ever deeper delving began into the unknown principles of maintenance of life as a phenomenon. 

Slowly, the growing lack of antibiotic effectiveness has led us along the path of learning about the mechanism of adaptation and even the evolution of the bacteria that carry the simplest forms of life, returning us to the very beginning and even simpler forms of life—viruses. Thus, the appearance of resistance in bacteria demonstrates one of the fundamental principles of preserving the phenomenon of life [9]. Thanks to technological advances, new ways of delivering antimicrobial peptides have been developed—one is by using nanoparticles, where those with silver are the best choice due to its antimicrobial activity [10,11,12,13]. 

This paper deals with some unconventional agents for treating bacterial infections caused by biofilm, in the light of the increasing bacterial resistance to antibiotics. The aim of the present review was to provide an overview of why bacterial viruses—bacteriophages and antimicrobial peptides are potential new agents in treating infections caused by resistant bacteria. 

## 2. Biofilm

Biofilm is one of the forms of bacterial adaptation that is increasingly leading to antibiotic resistance. Biofilm represents a crucial mechanism in the virulence and pathogenesis of medically significant bacterial pathogens (ESKAPE pathogens: *Enterococcus faecium, Staphylococcus aureus*, *Klebsiella pneumoniae*, *Acinetobacter baumanni*, *Pseudomonas aeruginosa*, and *Enterobacter* spp.) [14,15]. Antimicrobial therapy often becomes ineffective, precisely because of biofilm [16,17,18]. Biofilm is a sessile bacterial life form [19]. When bacteria integrate data from the environment, a mechanical and functional connection occurs. The presence of nutrients stimulates the expression of the genes associated with biofilm [20,21]. An extracellular matrix structure is formed, which consists of several types of extracellular polysaccharides, DNA, and proteins [16,22,23]. The biofilm channels allow the supply of nutrients, water, and air to each cell, giving it new properties—“multicellular” properties. It controls the population density by a signal mechanism from cell to cell known as quorum sensing, a process mediated by signal molecules called “autoinducers” [14,16,24]. When a particular biofilm density is reached at a critical concentration of autoinducers, the binding of these signal molecules to receptors leads to target gene repression. This modulation of control in the quorum sensing process allows the biofilm bacterial colony to maintain optimal size and encode virulent phenotypes [25,26,27,28]. This is also one of the characteristics of a multicellular organism.

The biofilm structure consists of a “skeleton” made up of exopolysaccharides, synthesized inside and outside the cell. Some of the exopolysaccharides are mannose, galactose, glucose, arabinose, fucose, rhamnose, xylose, galacturonic acid, glucosamine, and xylose. It has also been observed that exopolysaccharides synthesis is a result of reaction to stress when the bacterium is “attacked” by an antibiotic [29]. Some of these (mannose, rhamnose, glucose) promote the initial process of attachment of the bacteria to the substrate. Alginate is an exopolysaccharide associated with biofilms. It is not involved in biofilm initiation, but it is crucial in chronic infections because it protects the bacteria from antibiotics and represses host immune response [30]. In the production of alginate, there are 24 genes involved, while four genes and four proteins produce “intercellular glue” [30,31]. “Intercellular glue” is a linear polysaccharide composed of β-1,6-linked glucosamine residues [30]. Extracellular proteins help create and stabilize the biofilm, and amyloids play a supporting role in biofilm architecture [32]. Extracellular DNA plays a vital role in attaching the biofilm to the substrate. Its ability to chelate magnesium creates resistance to antimicrobial peptides, and inhibits the transport of antibiotics (vancomycin) [3,33,34], thus protecting the bacteria embedded in the biofilm. 

Biofilm antibiotic tolerance (BAT) is defined as the ability of bacteria living inside a biofilm to survive antimicrobial treatment using a set of genes [25,35,36]. Biofilm is a predominantly natural form of bacterial life because it increases their tolerance for challenging environmental conditions (avoiding flushing with water or blood). Bacteria inside biofilm are approximately 1000 times more resistant to antimicrobials than planktonic cells. Likewise, the biofilm protects the bacterial cells in the deeper layers from antimicrobial agents, and, due to the increased cell density, it facilitates the exchange of plasmid DNA through conjugation [30]. 

**Figure 1:**
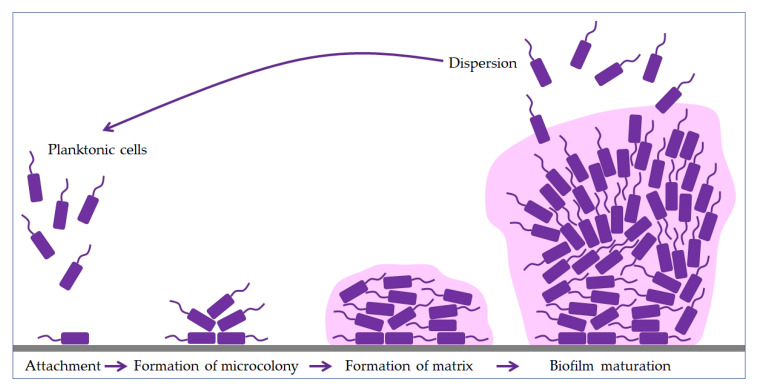
Phases of biofilm formation. The formation starts with the attachment of planktonic cells (purple), followed by binding to the surface (grey). The bacteria then form a microcolony and begin to produce an extracellular matrix (pink). In the maturation phase, the biofilm grows into a tower or mushroom-shaped structure due to the polysaccharides. Finally, some bacteria start to disperse to another site and form a new biofilm.

Bacteria use flagella and fimbriae to overcome the initial refusal to bind a negatively charged bacteria surface to an equally negatively charged environmental surface [30,37]. Five phases are essential for biofilm formation and maintenance: attachment, formation of a microcolony, formation of a matrix, maturation, and dispersion (Figure 1). After the phase of binding the first layer to the surface, the biofilm grows into a tower or mushroom-shaped structure in several hundred layers [16]. Anaerobic bacteria occupy deeper layers within the biofilm community, where they communicate and take on specific tasks. There is also a minute subpopulation of bacterial cells, called persister cells, which live in a dormant state and show extreme antimicrobial tolerance [25,38,39,40]. Studies have shown that persister cells are a phenotypic variant, not a mutation [16,30,40,41]. 

The biofilm contains increasing amounts of proteins, DNA, and polysaccharides secreted by trapped bacteria as it matures [42]. However, this is precisely why biofilm dispersion follows, which could lead to partial or complete biofilm degradation, but the planktonic cells thus created promote the formation of new biofilms.

## 3. Diseases Caused by Bacterial Biofilms 

Although most bacteria live in the form of biofilm, special attention is focused on clinically relevant bacteria that cause high mortality: ESKAPE (*Enterococcus faecium*, *Staphylococcus aureus*, *Klebsiella pneumoniae*, *Acinetobacter baumanni*, *Pseudomonas aeruginosa*, and *Enterobacter* spp.), but also many other pathogens such as *Proteus* spp. [15,43]. According to the National Institutes of Health (NIH), more than 80% of microbial infections are biofilm-related [25,44]. They show particular affinity in infections of wounds, lungs, the urinary system, joints, heart valves, teeth, and colonizations on medical implants and catheters are widespread [45,46,47,48]. Infections with resistant strains are common in cystic fibrosis, urinary tract infections [49], chronic wounds [50,51,52,53], prosthetic joint infections, prosthetic endocarditis, diabetic infections [51,54], and periodontal diseases [48].

The characteristic of all these infections caused by resistant strains of bacteria is the antibiotics’ inability to penetrate the pathogen due to the biofilm structure [55]. Exopolysaccharides and DNA significantly reduce antibiotic contact with the pathogen. This is especially evident when the antibiotics’ action should be most effective and is most necessary—in the phase of the exponential growth of the number of pathogens. The density of extracellular substances and the high concentration of bacterial agglomerations allow the enhanced transfer of plasmids and resistance genes (by conjugation and mobilization). Due to the limited and reduced availability of oxygen to cells in the biofilm’s deep layers, they reproduce slowly. Thus, they are still less sensitive to antibiotics, whose main form of action is blocking bacterial replication (beta-lactams) [44,50,56]. Efflux pumps, which are able to eject intracellular toxins due to the channels created, are also able to eject antibiotic drugs. The EPS matrix itself physically protects biofilm cells from nonspecific antibodies from leukocytes [57]. 

## 4. Biofilm Treatment Strategies 

The ability to make biofilm is an evolutionary achievement, with new “multicellular ability” traits that allow bacteria to survive, infect, multiply, and permanently infect hosts. As already mentioned, the resistance of clinically relevant bacteria (ESKAPE) and other groups of resistant bacteria is primarily the ability to infect the host despite biofilm inhibition measures taken, such as surface change and modification (medical implants or other biomaterials) using antibacterial agents, where the coating creates a barrier to bacterial adhesion [15,43,44]. In addition, the use of small molecules of bacterial biofilm inhibitors creates antifilm properties that passivate the surface of implants or medical devices (such as phenols, imidazoles, indole) [25,58,59]. An alternative method in biofilm control is the use of biologically active agents, such as a predatory bacteria species [60]. 

Biofilm dispersion is the second strategy in treating biofilm infections. More precisely, the disruption of quorum sensing by chemical means leads to biofilm dispersion [61,62,63]. However, biofilm dispersal agents should be combined with an antimicrobial agent [25,64]. Namely, if these dispersed bacteria are not treated simultaneously with antibacterial agents, they will inevitably form new biofilms by infecting new areas [36,44]. Treatment by co-administration of drugs and dispersal agents is very complex and challenging. However, as usual, the answer to this phenomenon already exists in microbiocenosis, by “infecting” the biofilm with viruses—bacteriophages. 

### 4.1. Bacteriophages

Bacteriophages are viruses that infect bacteria. Bacteriophages have been infectiously parasitic on bacteria from the very beginning of life. This virus–bacterium relationship is the oldest form of microbiocenosis, and perfection has been achieved in the form of a specific match between the virus –bacteriophage and the host cell-bacterium [65,66,67]. 

The first practical and positive experiences of using bacteriophages in controlling bacterial infections were developed during the Second World War. Bacteriophages found their application in treatment of war wound infections (explosive and blast injuries) before the use of antibiotics [68]. In extensive infections of such wounds, pathogen-specific bacteriophage preparations (*Pseudomonas aeruginosa*, *Staphylococcus aureus*, *Escherichia coli*, *Klebsiella* spp.) were applied directly to the site of infection (biofilm infections) [69]. 

**Figure 2:**
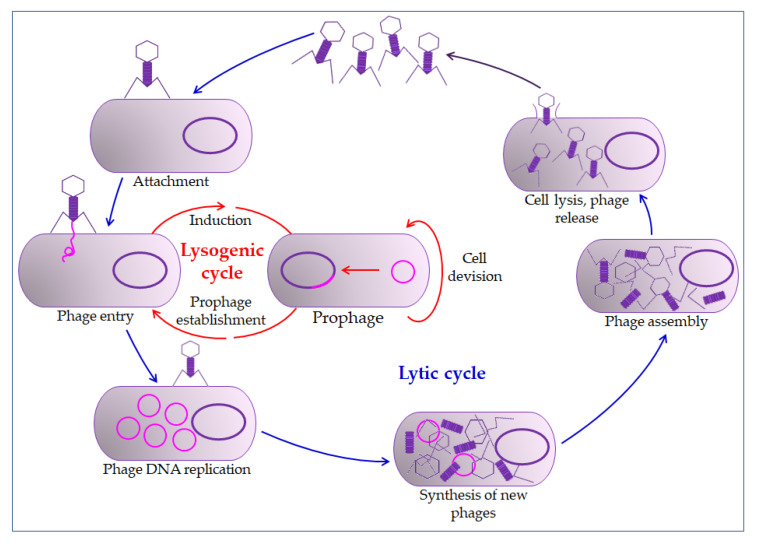
Bacteriophage life cycle. The bacteriophage first interacts with receptors on the host, absorbs, and then injects its genome to infect a bacterium. The lytic cycle involves the production of new bacteriophages and their release from the infected cell by lysis. The lysogenic cycle results in integrating a phage genome into the bacterial genome, which replicates in concert with the host DNA.

The mechanism of phage action on the prokaryotic cell begins with overcoming the cell membrane’s defense mechanisms, the incorporation of the genome into the cytoplasm, and the proliferation of phages. Phages impair the bacterial cells’ normal function by their proliferation and thus inactivate or kill cells (lysogenic or lytic cycle, Figure 2) [65,68]. The relationship between bacteriophages and bacteria in biofilm is far more complex. Bacteriophages must have the ability to encode a depolymerase that degrades the biofilm matrix, which includes degradation of polymers, capsular polysaccharides, and extracellular DNA [70,71]. Only then do they access cell membranes and receptors. In the treatment of bacterial infections, the addition of several enzymes that increases the activity of phages is used, which leads to synergistic removal of bacteria. Thus, it is essential to create the conditions to bypass the bacterial biofilm matrix [72,73]. Therefore, bacteriophages are able to penetrate the membrane receptors, but antibiotics cannot due to the biofilm’s defense mechanisms [51,74]. The combination of phage and antibiotics seems to be the optimal combination in the fight against biofilm. In most cases, it is optimal in various combinations, even in combination with disinfectants [75,76]. However, in some situations, phage application could even lead to enhanced bacteria aggregation in the biofilm, surface adhesion, and fimbriae production. This usually occurs in Gram-negative bacteria leading to inhibition of phage penetration through the biofilm [72,77,78].

Furthermore, there are circumstances in which phages stimulate biofilm formation, such as increased bacteriophage pressure in the biofilm, leading to larger aggregates, which could be considered as an evolutionary adaptation [72,77]. Thus, such interaction between bacteriophage and the host could be classified as mutualistic rather than parasitic. In so doing, bacteriophages acquire some new properties which are favorable to them, such as encapsulation in the biofilm matrix, in which phages can tolerate higher concentrations of disinfectants, radiation, and other environmental factors [51,72].

All this points to the need for careful preparation before the application of bacteriophages. Due to prophage induction, extracellular DNA accumulates in the biofilm [72,79]. Although they could lead to cell death, prophages are integrated phages in the host’s lysogenic cycle. They may encode virulence factors and antibiotic resistance factors (toxins, enzymes, and superantigens) in cholera, for example [72]. 

Bacteriophage evolution stimulated by phage and host interaction in the biofilm, potentiates and promotes mutations as common properties. It could demonstrate the real potential of bacteriophage therapy to eradicate infectious biofilms [76,80]. Thus, hundreds of years of positive practical experience in the application of bacteriophages, their easy isolation, cost-effectiveness, absolute specificity to the host, self-reproduction, and non-disruption of normal microbiocenosis, without harmful side effects, make bacteriophages the choice for the future [65,81].

There are a few more potential benefits of bacteriophages. The ability to “deliver” broad-spectrum antimicrobial drugs to the infection site makes bacteriophages extremely potent in creating even more effective modular antibacterial agents [82]. Another particular interest is the concept of enhancing the phage genome to express antimicrobial peptides (AMPs) [15]. 

### 4.2. Antimicrobial Peptides (AMPs)

Biofilm eradication agents (BEAs) are the target of many modern studies, and antimicrobial peptides (AMPs) are among the most likely BEAs [25,83,84,85]. AMPs are ubiquitous compounds produced by plants, invertebrates, and animals [86], and are relatively simple molecules (from 5 to 100 amino acids) with a molecular mass of 1–5 kDa [15,87]. They are predominantly cationic, so they are also called cationic antimicrobial peptides. The mechanism of their antimicrobial action is associated mainly with cytoplasmic membrane disorder, and inhibition of protein accumulation or enzymatic activity. In eukaryotes, they play an essential role in innate immunity [57,88]. 

Plants and invertebrates lack adaptive immunity (immunity mediated by B and T cells), so AMPs play a fundamental role in protecting against bacterial and fungal infections [86]. All plant AMPs are rich in cysteine and contain many disulfide bonds. In invertebrates, AMPs are found in hemolymph, hemocytes, phagocytes, and epithelial cells [86,87]. The vertebrate immune system consists of an innate and adaptive immune system. AMPs can be isolated from leukocytes, phagocytes, epithelial macrophages, and body fluids [87,89,90,91]. The most prominent groups of mammalian AMPs are cathelicidins and defensins [57,92]. 

AMPs are classified according to structure, sequence, or mechanism of action. AMPs may have several activities: bactericidal, immune modulations, antiviral properties, anticarcinogenic properties, and they can prevent biofilm formation. Since AMP activity depends on their structure and sequence, it is crucial to consider both properties when categorizing them [86,87]. 

#### 4.2.1. The Mechanism of Action of Antimicrobial Peptides

The mechanism of action of AMPs can be divided into two groups: the direct killing of microorganisms (by membrane targeting or non-membrane targeting), or immune modulation [15,88,93]. 

The permeabilizing membrane mechanism of action may have receptor- or non-receptor-mediated interactions. Some AMPs, such as nisin, bind with high affinity to lipid molecules in the cell membrane, producing pores in it, and act by covering the entire surface of the membrane, the so-called carpet model (detergent-like model) [62,86,94]. 

**Figure 3:**
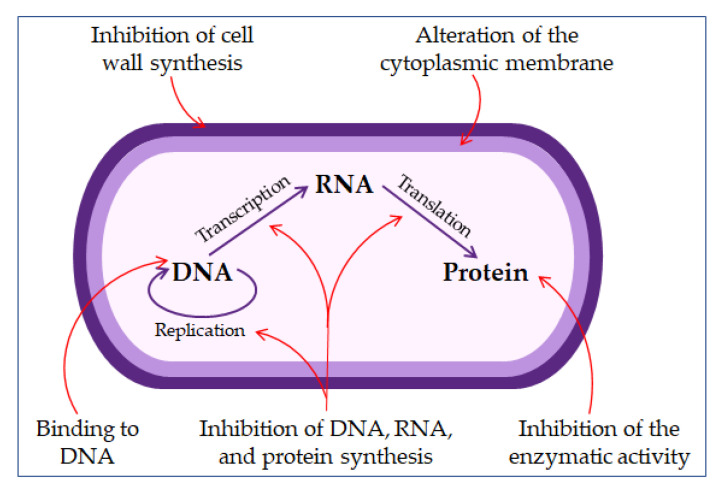
The mechanisms of antimicrobial peptides action.

The direct non-membrane targeting mechanisms of action are based on AMPs targeting the bacterial cell wall to inhibit cell wall synthesis. AMPs interact with the diverse precursors needed for cell wall synthesis. For example, AMP defensins bind to the charged pyrophosphate sugar moiety of the lipid molecule [86,95]. While some AMPs can act on the cytoplasmic membrane, others accumulate in the cytoplasm and inhibit the synthesis of proteins and nucleic acid, thus disrupting enzyme–protein activity (Figure 3) [86,96]. 

The mechanisms of action of AMP in immune modulation include various immune responses. Immune cells (neutrophils, macrophages) produce AMPs, which are the first to contact the microbial invasion [97,98]. Likewise, AMPs promote a diversity of immune responses: activation, attraction, and differentiation of leukocytes. Some human AMPs (LL-37, β defensins) can attract immune cells, such as white blood cells, dendritic cells, and mast cells [86,99,100,101]. Some research suggests that AMPs might serve as vaccine adjuvants [86].

The properties of AMPs can be improved to enhance their delivery, by optimizing their stability and toxicity. This is mainly done through polymer conjugation: conjugation with biopolymers such as chitosan and hyaluronic acid. Alternatively, it may be done by encapsulating AMPs in micelles and liposomes [86,102]. 

Antiviral AMPs can neutralize the virus by integrating into the viral envelope and cell membrane, causing destabilization of the viral membrane or preventing the host’s infection [99]. Antiviral AMPs defensins can bind to viral glycoprotein, after which viruses (such as herpes simplex virus) are unable to bind to the surface of the host cell [92]. Some AMPs (such as lactoferrin) can occupy specific mammalian cell receptors and prevent the virus from binding to its target receptor (such as heparan molecules for herpes simplex virus), and blocking viral interaction with the receptor [85,87]. Some antiviral AMPs can enter the host cell itself where they are located in the cytoplasm or the organelles and alter the host cell gene expression, thus helping the host defense mechanism [85,103,104]. 

Thus, AMPs, with all their properties and mechanisms of action (structural, therapeutic), are incredibly suitable molecules in the treatment, especially of infections resistant to many drugs (mainly resistant to antibiotics) [105,106,107,108]. All future research should aim at discovering the improvement of AMPs intake and their action, and their action combined with other antimicrobial agents (antibiotics, bacteriophages) [109,110,111]. This mainly refers to their biocompatibility action in the immunomodulation system [84,101,107]. It is also necessary to avoid undesirable consequences of AMP administration such as toxicity, hemolytic activity, and changing their structure, primarily of cationic AMPs, to obtain even more efficient and safer AMPs [108].

#### 4.2.2. The Benefit of Combined Therapy of Antimicrobial Peptides and Nanoparticles

New AMP delivery systems are being developed, which could help avoid the problems related to AMP delivery [15,112]. They improve the pharmacokinetics of AMPs [10], increase their half-life, reduce the required dose, and decrease production costs and possible toxicity [10,11,113]. All this may be achieved by encapsulating AMPs in various nanocarriers [15,112]. Nanoparticles significantly increase the penetration of AMPs into cells [45,113]. 

Several metal nanoparticles, such as silver and gold, have appeared as a possible choice for treating antibiotic-resistant bacterial infections [12]. Silver nanoparticles (AgNPs) are particularly interesting because they have potent antimicrobial activity [12,114]. Both AgNPs and AMPs could replace antibiotics, and the conjugation of AMPs with AgNPs has the additional advantage of the synergistic effect of their antimicrobial properties [115,116,117]. The combination of AMPs and AgNPs might produce new features, such as higher antibacterial activity, increased stability, reduced toxicity, and enhanced selectivity [13,118,119].

Combined therapy using AgNPs and AMPs represents a new approach in the development of new antimicrobial drugs [120]. 

## 5. Conclusions

Today’s findings on microbial diseases (primarily bacterial) indicate the constant dynamic of microorganisms in adaptation to antimicrobial drugs. The most harmful and clinically significant pathogens are classified in resistant groups (ESKAPE, MRSA, VRE) and create a biofilm as a biological response to drugs. They acquire various forms of resistance by rapid mutations, changes in antigen structure, and adapt their mechanisms of virulence and contagiousness. By studying these models, mechanisms, and principles, treatment options arise from the microorganisms’ environment. One is the use of the evolutionary abilities of some other microorganisms. Thus, bacterial antagonists, bacteriophages, and their infection mechanisms and parasitism on bacteria are used to improve the treatment of severe infections.

This principle that nature offers solutions exactly where problems arise is combined with increasing knowledge about relatively simple proteins. AMPs are able to act alone or in combination with known or innovated antibiotics, bacteriophages, and nanoparticles. AMPs open up many new beneficial possibilities in treating severe and deadly infections, and even malignant diseases. It is precisely this knowledge that is increasingly growing about yet undiscovered immune functions. It is also essential in discovering other possibilities of the human genome in creating more comfortable, good quality, and longer life.
